# Trunk Flexion Monitoring among Warehouse Workers Using a Single Inertial Sensor and the Influence of Different Sampling Durations

**DOI:** 10.3390/ijerph17197117

**Published:** 2020-09-28

**Authors:** Micaela Porta, Massimiliano Pau, Pier Francesco Orrù, Maury A. Nussbaum

**Affiliations:** 1 Department of Mechanical, Chemical and Materials Engineering, University of Cagliari, 09123 Cagliari, Italy; massimiliano.pau@dimcm.unica.it (M.P.); pforru@unica.it (P.F.O.); 2 Department of Industrial and System Engineering, Virginia Tech, Blacksburg, VA 24061, USA; nussbaum@vt.edu

**Keywords:** inertial measurement unit (IMU), manual material handling task (MMH), trunk flexion

## Abstract

Trunk flexion represents a risk factor for the onset of low-back disorders, yet limited quantitative data exist regarding flexion exposures in actual working conditions. In this study, we evaluated the potential of using a single inertial measurement unit (IMU) to classify trunk flexion, in terms of amplitude, frequency, and duration, and assessed the influence of alternative time durations on exposure results. Twelve warehouse workers were monitored during two hours of an actual shift while wearing a single IMU on their low back. Trunk flexion data were reduced using exposure variation analysis integrated with recommended exposure thresholds. Workers spent 5.1% of their working time with trunk flexion of 30–60° and 2.3% with flexion of 60–90°. Depending on the level of acceptable error, relatively shorter monitoring periods (up to 50 min) might be sufficient to characterize trunk flexion exposures. Future work is needed, however, to determine if these results generalize to other postural exposures and tasks.

## 1. Introduction

Non-neutral trunk postures, in particular those involving flexion [1], represent a risk factor for the onset of low-back disorders [2,3,4,5]. These disorders represent a major health problem, causing absence from work, with consequent reduction in productivity [6], and in the most serious cases disability and impairment of the fundamental activities of daily living. Such adverse outcomes, along with their associated social costs [7], have led ergonomists and safety professionals to try to improve job design decisions, and to establish suitable safe work limits that either prevent these outcomes or, at least, mitigate their adverse effects.

A critical issue when planning prevention/mitigation strategies is obtaining an accurate evaluation of the biomechanical risk associated with each working task, which requires a detailed identification of task intensity, frequency, and duration [8]. Traditionally, such assessments have been typically performed by means of self-reports and observational methods. The former method is easy to use and inexpensive, but can be biased by subjective perceptions of physical work demands, while the latter can be inaccurate and substantially time-consuming, with required analysis durations up to 30 times the actual duration of a video segment [9].

In the last three decades, however, several quantitative techniques have been developed and used to obtain direct measurements of trunk postures under actual working conditions, including electro-goniometers [10] and inclinometers [11]. Unfortunately, some of these instruments are not suitable for long-term measurements, since they require additional external structures to be connected to the body. Since they are often attached on the subject’s skin, they can also cause discomfort [12]. Moreover, these devices can alter an individual’s usual working behaviors.

In recent years, the rapid advancement of technology and a parallel reduction in cost has made it easier to employ substantially smaller devices, such as wearable inertial measurement units (IMUs), to obtain quantitative data on movement kinematics. Such devices have rapidly become popular among human movement researchers, as they provide reliable data, with quality comparable to gold standards (i.e., optical motion capture systems: [13,14,15,16,17,18]). IMUs represent an appealing option even for field-based settings, as they do not interfere with worker’s task and can acquire a large amount of data, thus being suitable for long-term posture monitoring.

For lumbar posture, the most common parameter in assessing exposure is the angular position of the trunk, commonly expressed using an ordinal scale (e.g., neutral, mild, and severe flexion), even when continuous data are recorded. In most cases emphasis is given to the level of exposure neglecting both frequency and duration, although duration of exposure may be used to evaluate cumulative exposure proportional to chronic damage [8].

In the last decade, several reports have indicated that IMUs can effectively monitor body posture during the performance of diverse tasks, both in laboratory conditions [13,18,19,20,21,22,23,24] and in situ [6,18,25,26,27,28,29,30]. However, while these have investigated trunk kinematics, no information was provided about critical aspects of postural exposure, such as the frequency and duration of trunk movements.

Assessing trunk posture is of particular interest among warehouse workers. These workers are often assigned to order-picking processes, in which they perform a variety of physically-demanding tasks, such as restocking shelves and pallets, loading and unloading pallets, and driving forklifts. In many warehouse facilities, it is typical for a worker to spend the majority of their shift picking items from shelves and stacking them onto a pallet to form orders that are then shipped. Since such tasks are not easily automatable, they are still mostly performed manually, with limited (or no) mechanical support. As such, warehouse workers commonly perform substantial manual material handling (MMH) tasks that involve repetitive exposures to non-neutral trunk postures, and experience a relatively high rate of low-back disorders [31,32,33].

In such a context, IMUs can be considered a valid option to monitor several aspects of movements associated with working tasks, as their interference with regular movements appears negligible and thus their use seems acceptable in workplaces. However, issues related to compliance, protection of personal data, and comfort, as well as the lack of standardized protocol for data acquisition and processing, are still under debate. Thus, the use of such devices in daily practice is not as widespread as expected [34,35,36].

To help overcome some of these concerns, we report here on a study that assessed the feasibility of classifying trunk flexion during actual MMH tasks among warehouse workers using a miniaturized wearable IMU. The main purpose of the study was to determine the potential of a simplified setup, a single IMU, suitable for application in actual working conditions for long-term monitoring, and to provide a reliable classification of trunk postures in terms of frequency and duration. Additional analysis was completed to investigate the influence of the monitoring duration, to explore possible efficiencies in data collection that might help toward improving worker acceptability and enhancing the number of workers that can be included in exposure assessments.

## 2. Materials and Methods

### 2.1. Participants

Twelve male, full-time workers participated voluntarily, with mean (SD) age = 35.4 (9.1) years, height = 172.7 (5.3) cm, body mass = 72.3 (12.6) kg, and seniority in service = 9.5 (6.2) years. Each participant was employed at the main regional warehouse in Sardinia of “Conad del Tirreno Soc. Coop. Srl” (the largest Italian retail supplier). At the time of the study, all workers were free from any signs of acute or chronic musculoskeletal conditions and, after a detailed explanation of the purposes and methodology of the study, signed an informed consent form. The study was carried out in compliance with the ethical principles for research involving human subjects expressed in the Declaration of Helsinki and its later amendments. Participants were routinely assigned to the same kind of tasks during a regular shift, namely: (1) refilling shelves with small packages by moving goods from a vertical closet to roller units placed at variable heights; and (2) assembling orders to be delivered to local stores, following instructions received continuously via radio on the type and quantity of the necessary goods that will be subsequently placed on a pallet. Preliminary observations, along with interviews with the operators and their supervisors, indicated that the tasks completed by the workers exhibited distinct characteristics of cyclicity and repeatability. Such aspects were considered to select the monitoring duration in the experimental tests, as described in detail below.

### 2.2. Experimental Protocol

Trunk posture was monitored continuously for two hours, randomly chosen, during a regular 8-h work shift, using a lightweight miniaturized IMU (G-Sensor, BTS Bioengineering S.p.A., Garbagnate Milanese, MI, Italy) that includes tri-axial accelerometers and gyroscopes, and a magnetometer. The IMU was placed on the lower back using a semi-elastic belt, at roughly three-quarters of the distance from the C7 vertebrae to the mid-point between posterior superior iliac processes, according to the approach described by Faber et al. [13], which indicated this placement as optimal to investigate trunk inclination during symmetric and asymmetric lifting.

### 2.3. Trunk Posture Acquisition and Processing

Raw accelerations and angular velocities were recorded onboard the IMU at 100 Hz and preprocessed by a Digital Motion Processor (DMP^TM^) (InvenSense Inc., San Jose, CA, USA), which provided rotational angles (i.e., roll, pitch, and yaw). Such data must be further processed to obtain Cardan angles referred to a global reference system. Before data acquisition, during the working tasks, the physiological trunk position (expressed in terms of flexion angle) was assessed by having participants stand for 10 s in a neutral, upright posture. This procedure supported removing subject-specific angular offsets and errors caused by sensor placement. Cardan angles about the IMU axes were then calculated using the YZX sequence (i.e., flexion-extension around Y; lateral bending around Z axis; axial rotation around X axis) as generalized by Cole [37].

Data processing was carried out by means of a custom routine developed in Matlab (R2019a, MathWorks, Natick, MA, USA) to classify trunk flexion angles as follows [30,38,39]:

- Class 1: flexion angle = 30°–60°
- Class 2: flexion angle = 60°–90°
- Class 3: flexion angle > 90°

We subsequently determined the duration of exposure to each posture class using an approach based on exposure variation analysis (EVA, [40]), which has been applied in previous work to investigate trunk posture in workplaces [41], with specific time periods of 0–2 s, 2–4 s, and >4 s. Finally, we assessed the time spent in each of the combinations of posture and time period classes in terms of either frequency or percentage of the total working time. Data were processed for the entire 2-h period.

**Figure 1:**
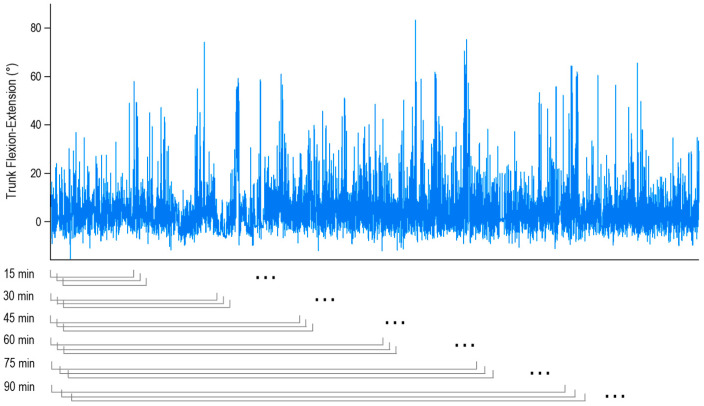
Example of two hours of trunk flexion data obtained from a participant (top). As shown at the bottom, windows of different duration (15, 30, 45, 60, 75, and 90 min) were moved across the entire signal, each in one-minute steps. Positive values indicate trunk flexion, while negative values indicate extension.

These data were also processed separately for different sampling durations, to explore the effects associated with possible reductions of the monitoring time. To do so, we considered moving windows of different durations (from 15 to 90 min, in 15 min increments), each at one-minute steps over the full two-hour sample (Figure 1). Mean values of the percentages spent in each class of flexion obtained for each duration were compared with those calculated using the original 2-h sample (considered as the “true” value).

**Figure 2:**
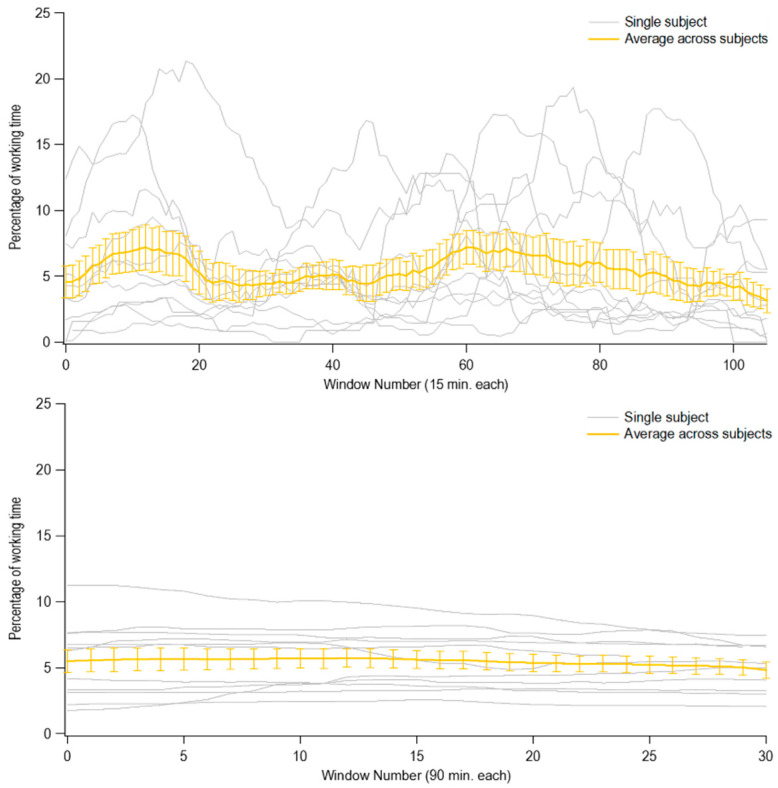
Examples of averages calculated across subjects using two different window durations. Grey curves represent the percentage of time in Class 1 trunk flexion for each subject, obtained with moving windows of 15 min (top) and 90 min duration (bottom), each at one-minute steps across the full two-hour sample. Yellow curves represent averages (standard errors) calculated step-by-step across subjects.

**Figure A1:**
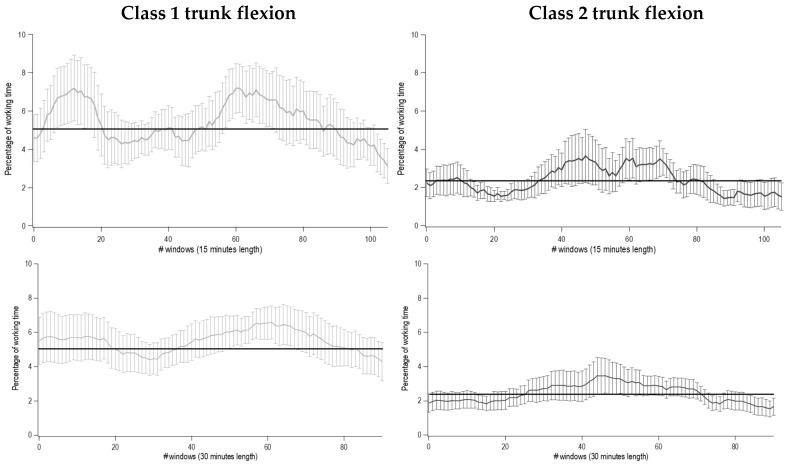

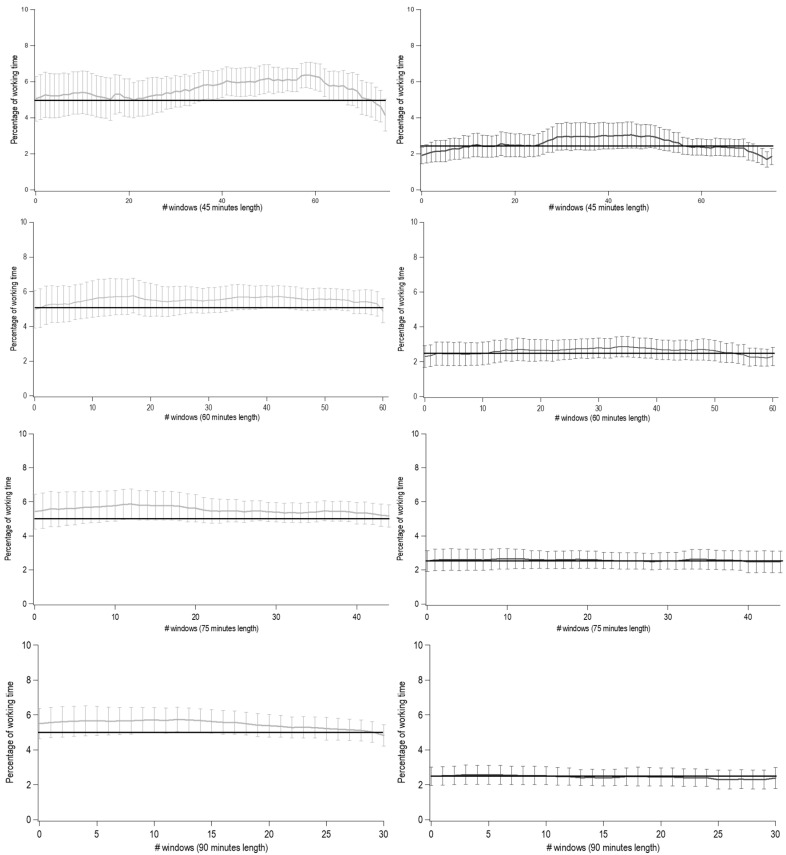
Mean values of the percentage of time spent in two classes of trunk flexion. Each point represents the mean value (standard error) calculated across the workers using windows of different duration, from 15 (top) to 90 (bottom) minutes, using steps of 1 min. Black lines represent the “True” value calculated as mean exposure obtained from the entire 2 h of monitoring.

A relative mean squared error (MSE) was calculated to capture the error associated with a specific window duration. Figure 2 provides two examples (using the shortest and longest durations) for Class 1 flexion, demonstrating how averages across the subjects have been calculated (Figure A1 in the Appendix A shows results obtained for each class of flexion and for each of the six window durations).

## 3. Results

**Figure 3:**
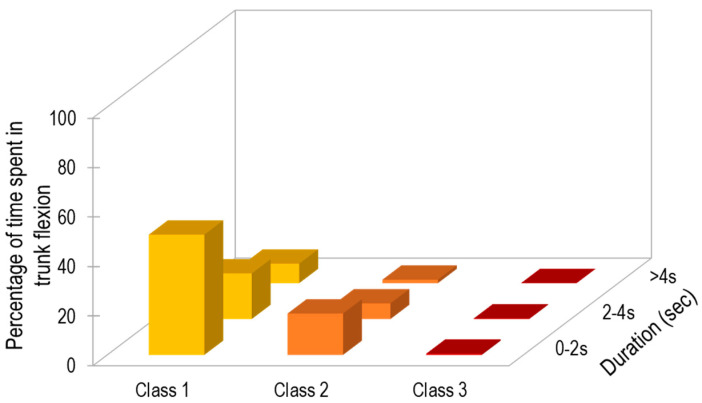
Results from exposure variation analysis of trunk flexion. Bars represent the percentage of time spent in different categories of trunk flexion, based on flexion amplitude and duration. This representation captures the distribution of flexion pattern (e.g., nearly 70% of flexed posture occurred for less than two seconds and most trunk flexion was between 30° and 60°).

Table 1 summarizes the working time spent in trunk flexion according to the three defined classes, based on the full acquisition period (i.e., 2 h). Participants spent 5.1% of the time in trunk flexion between 30° and 60°, and 2.3% of the time with flexion between 60° and 90°. The percentage of time spent in postures characterized by angles >90° was negligible (0.07%). Results of the EVA analysis are shown in Figure 3. Considering only the angles higher than 30 degrees (which represent approximately 8% of the monitored time, the majority (~70%) of flexion angles ranged between 30 and 60° (i.e., Class 1), while lower percentages were observed for Class 2 (28%) and Class 3 (<1%) flexion. In terms of event duration, most (60%) trunk flexion events lasted ≤2 s, 24% had a duration of 2-4 s, and <10% were maintained for >4 s.

**Figure 4:**
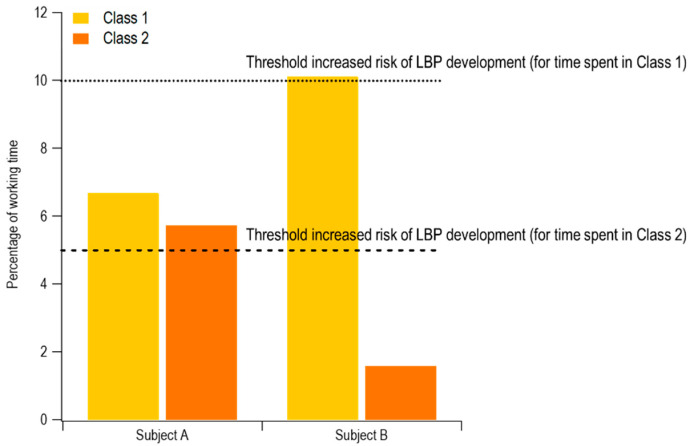
Example of different postural behaviors adopted by two participants performing the same kind of activities. Noted thresholds for low back pain (LBP) are from [38].

Although at the group level the percentage of time spent in trunk flexion appeared relatively low, analysis at the individual level revealed different behaviors, as shown in Figure 4. For example, participant A spent 6.7% of the time with trunk flexed between 30° and 60°, and 5.7% of the time with trunk flexed between 60° and 90°. In contrast, participant B spent 10.1% with trunk flexion between 30° and 60° and only 1.6% of the time with trunk flexion between 60° and 90°. In both cases, the obtained values exceeded those recommended to avoid an increased risk of low back pain [38].

**Figure 5:**
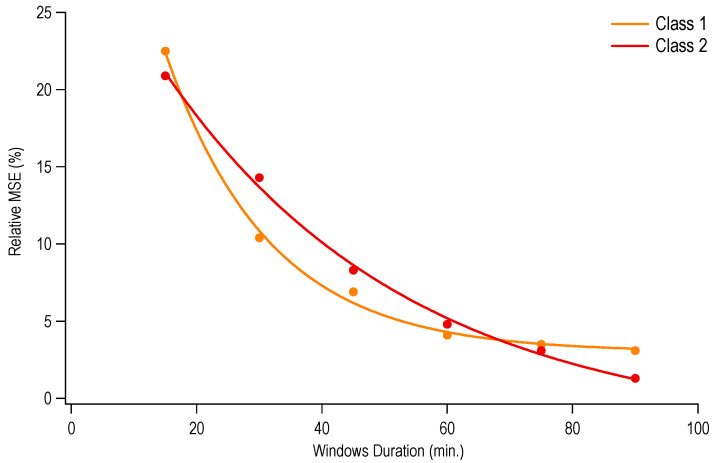
Relative mean squared error (MSE), indicating the difference between mean values of the time spent in trunk flexion and the mean “True” value (from the full 2-h sample). Values are given for several window durations (i.e., 15, 30, 45, 60, 75, and 90 min). Exponential curve fits are indicated by solid lines.

Relative MSE values, capturing differences obtained with reduced monitoring durations with respect to the 2-h value, are shown in Figure 5. This error appears to decrease exponentially with increasing window duration. However, MSE values were quite similar for windows durations ranging from 60 to 90 min (between 4.1% and 3.1% for Class 1, and between 4.8% and 1.3% for Class 2).

## 4. Discussion

The main purpose of the present study was to assess the feasibility of using a simplified setup involving a single miniaturized wearable IMU to characterize a worker’s exposure to trunk flexion. The proposed approach is potentially suitable for long-term monitoring under actual working conditions, since it is based on a sensor that is of limited size, is easily positioned, and provides data with a straightforward interpretation. We tested this setup using a sample of warehouse workers, during the execution of tasks characterized by a marked cyclicity for 2-h of an actual shift; this approach may be suitable for application to other tasks characterized by similar features.

IMU data on trunk flexion were reduced using the EVA approach integrated with recommended exposure thresholds. Overall, the current participants spent 5.1% of their working time with trunk flexion of 30–60°, and 2.3% of their time with trunk flexion 60–90°, while the time spent with trunk flexion exceeding 90° was negligible. In their 3-year prospective studies, Hoogendorn et al. [38] and Coenen et al. [42] assessed physical load in terms of trunk postures and the number of lifts by means of video analysis. They estimated that workers who spend either more than 10% of their daily shift with trunk flexion exceeding 30°, or more than 5% of the time with trunk flexion exceeding 60°, are at an increased risk of the onset of low back disorders. In contrast, no explicit conclusions were formulated for flexion exceeding 90°. However, it is reasonable to assume that such extreme postures will be associated with an increased risk of low back disorders, given that trunk flexion amplitude is positively correlated with the load sustained by the intervertebral disks [43]. The patterns of trunk flexion found here are comparable with those reported by Jansen et al. [41] from a sample of nurses (who spent approximately 9% of the working time with the trunk flexed >30°) and housekeepers (10% of the working time with the trunk flexed > 30°). Similar trends were also reported by Jakobsen et al. [30] for warehouse workers and machinery operators. Although the workers tested here spent, on average, only 7.4% of the working time with their trunk flexed, it is noteworthy that most of these flexions were in the shortest duration category (duration < 2 s, see Figure 3), suggesting that the tasks required relatively rapid activities.

As a group, the tested participants would thus appear to be at low risk based on their trunk flexion exposures. However, for two participants the above-mentioned thresholds were exceeded. This is not surprising, since workers assigned to the same task can adopt distinct postural strategies according to factors such as experience, anthropometry, and optimization of energy expenditure [44,45,46]. Thus, the proposed simplified approach might be useful both to identify strenuous tasks and activities in a particular job (at the group level), and to detect potentially critical conditions that may exist even in presence of planned working conditions and suitable training programs, being applicable to a large sample of workers without a significant increase in measurement efforts. Application of the EVA approach [40] supports determining how different classes of trunk flexion are distributed as a function of their duration. Such information may also be useful to reproduce more realistic conditions for laboratory simulations, where the pace of the activity is usually determined a priori, along with the distribution and duration of different levels of trunk flexion.

One practical issue in the use of sensors in monitoring working postures (and other outcomes) is determining the duration of monitoring. While reducing the monitoring time would allow testing more workers in a given period and would decrease the degree of interference of measurements with the task, such reductions may be possible only when the working task is characterized by substantial repeatability. If so, once data are obtained for a complete working shift (such as during a characterization study), further periodic monitoring could be shortened without any substantial loss of information. We found that errors (relative MSE) decrease non-linearly with an increasing monitoring window duration (Figure 5). Although further quantitative investigations with larger samples are needed, our results suggest that 50–60 min periods of monitoring might be sufficient to capture postural exposures without substantial bias for the current task.

Some limitations in the current study should be acknowledged. First, we only analyzed postural exposure (specifically trunk flexion), although it is known that several other kinematic and kinetic aspects of exposure (e.g., movement velocity and external loads) are important in assessing biomechanical risk. In particular, velocity has been found [47] to be a stronger predictor of risk among trunk kinematic factors. IMUs are suitable to provide such information easily, since angular velocities are immediately available as raw output data. However, as pointed out by Burdorf and van Riel [8], posture is the basic element with which the other factors integrate to yield a complete picture of a worker’s exposure. Second, we only analyzed trunk flexion, even though it would be of interest to consider other trunk movements, such as combined flexion–rotation or flexion-lateral bending, as these are also implicated in the risk of onset of low back disorders [38]. Third, although our results suggest that 50–60 min of monitoring among warehouse workers might be sufficient to characterize the task in terms of trunk flexion, further studies are necessary to increase the generalizability of these results to other tasks. Finally, it would be interesting to explore the relationship between individual physical aspects, in terms of trunk range of motion and movement strategies adopted during the working shift, to understand whether limitations/impairments may influence a given workers material handling behaviors. Despite such limitations, the methodology proposed here may be useful especially for rapid in situ screenings of large cohorts of workers, while at the same time ensuring minimal disturbances to the working tasks.

## 5. Conclusions

In summary, the results here demonstrated how a simple, non-invasive setup using a single IMU can support the detection and classification of non-neutral trunk posture exposure in warehouse activities. Such an approach provides data regarding the amplitude, duration, and frequency of trunk flexion, and overcomes several typical limitations inherent in observational methods. Although further studies on larger cohorts are needed to confirm the findings here, especially as regards the selection of a specific monitoring duration, this approach appears promising and potentially suitable for diverse MMH tasks in which trunk flexion represents a critical component of biomechanical risk.

## Figures and Tables

**Table 1:** Results regarding different classes of trunk flexion. For each class of flexion, means (SD) of the percentage of time spent in different classes of trunk flexion are given from the entire 2-h observation periods and from several shorter window durations.

Windows Duration	Class 1 (30–60°)	Class 2 (60–90°)	Class 3 (>90°)
	Mean (SD)	Mean (SD)	Mean (SD)
2 h	5.1 (2.2)	2.3 (1.5)	negligible
90 min	5.3 (2.2)	2.4 (1.6)	-
75 min	5.4 (2.4)	2.5 (1.6)	-
60 min	5.3 (2.6)	2.5 (1.8)	-
45 min	5.2 (3.0)	2.5 (1.8)	-
30 min	5.3 (3.4)	2.4 (2.0)	-
15 min	5.3 (4.2)	2.4 (2.5)	-

## References

[1] Wai E.K., Roffey D.M., Bishop P., Kwon B.K., Dagenais S. (2010). Causal assessment of occupational bending or twisting and low back pain: Results of a systematic review. Spine J..

[2] Andersen J.H., Haahr J.P., Frost P. (2007). Risk factors for more severe regional musculoskeletal symptoms: A two-year prospective study of a general working population. Arthritis Rheum..

[3] Da Costa B.R., Vieira E.R. (2009). Risk factors for work-related musculoskeletal disorders: A systematic review of recent longitudinal studies. Am. J. Ind. Med..

[4] Bernard B.P. (1997). Musculoskeletal Disorders and Workplace Factors. A Critical Review of Epidemiologic Evidence for Work-Related Musculoskeletal Disorders of the Neck, Upper Extremity, and Low Back.

[5] Punnett L., Fine L.J., Keyserling W.M., Herrin G.D., Chaffin D.B. (1991). Back disorders and nonneutral trunk postures of automobile assembly workers. Scand. J. Work. Environ. Health.

[6] Villumsen M., Samani A., Jørgensen M.B., Gupta N., Madeleine P., Holtermann A. (2014). Are forward bending of the trunk and low back pain associated among Danish blue-collar workers? A cross-sectional field study based on objective measures. Ergonomics.

[7] Dunning K., Davis K.G., Cook C., Kotowski S.E., Hamrick C., Jewell G., Lockey J. (2009). Costs by industry and diagnosis among musculoskeletal claims in a state workers compensation system: 1999–2004. Am. J. Ind. Med..

[8] Burdorf A., Van Riel M. (1996). Design of strategies to assess lumbar posture during work. Int. J. Ind. Ergon..

[9] Heberger J., Nasarwanji M., Paquet V., Pollard J.P., Dempsey P.G. (2012). Inter-Rater Reliability of Video-Based Ergonomic Job Analysis for Maintenance Work in Mineral Processing and Coal Preparation Plants. Proc. Hum. Factors Ergon. Soc. Annu. Meet..

[10] Marras W., Fathallah F., Miller R., Davis S., Mirka G. (1992). Accuracy of a three-dimensional lumbar motion monitor for recording dynamic trunk motion characteristics. Int. J. Ind. Ergon..

[11] Williams R., Binkley J., Bloch R., Goldsmith C.H., Minuk T. (1993). Reliability of the Modified-Modified Schöber and Double Inclinometer Methods for Measuring Lumbar Flexion and Extension. Phys. Ther..

[12] David G. (2005). Ergonomic methods for assessing exposure to risk factors for work-related musculoskeletal disorders. Occup. Med..

[13] Faber G.S., Kingma I., Bruijn S.M., Van Dieën J.H. (2009). Optimal inertial sensor location for ambulatory measurement of trunk inclination. J. Biomech..

[14] Goodvin C., Park E.J., Huang K., Sakaki K. (2006). Development of a real-time three-dimensional spinal motion measurement system for clinical practice. Med. Biol. Eng..

[15] Kim S., Nussbaum M.A. (2013). Performance evaluation of a wearable inertial motion capture system for capturing physical exposures during manual material handling tasks. Ergonomics.

[16] Lebel K., Boissy P., Nguyen H.P., Duval C. (2017). Inertial measurement systems for segments and joints kinematics assessment: Towards an understanding of the variations in sensors accuracy. Biomed. Eng. Online.

[17] Robert-Lachaine X., LaRue C., Denis D., Delisle A., Mecheri H., Corbeil P., Plamondon A. (2019). Feasibility of quantifying the physical exposure of materials handlers in the workplace with magnetic and inertial measurement units. Ergonomics.

[18] Schall M.C., Fethke N.B., Chen H., Oyama S., Douphrate D.I. (2015). Accuracy and repeatability of an inertial measurement unit system for field-based occupational studies. Ergonomics.

[19] Faber G.S., Chang C., Kingma I., Dennerlein J.T., Van Dieën J.H. (2016). Estimating 3D L5/S1 moments and ground reaction forces during trunk bending using a full-body ambulatory inertial motion capture system. J. Biomech..

[20] Cutti A.G., Giovanardi A., Rocchi L., Davalli A., Sacchetti R. (2007). Ambulatory measurement of shoulder and elbow kinematics through inertial and magnetic sensors. Med. Biol. Eng..

[21] Godwin A., Agnew M., Stevenson J. (2009). Accuracy of Inertial Motion Sensors in Static, Quasistatic, and Complex Dynamic Motion. J. Biomech. Eng..

[22] Kim S., Nussbaum M.A. (2014). An evaluation of classification algorithms for manual material handling tasks based on data obtained using wearable technologies. Ergonomics.

[23] Lim S., D’Souza C. (2019). Statistical prediction of load carriage mode and magnitude from inertial sensor derived gait kinematics. Appl. Ergon..

[24] Yan X., Li H., Li A.R., Zhang H. (2017). Wearable IMU-based real-time motion warning system for construction workers’ musculoskeletal disorders prevention. Autom. Constr..

[25] Arias O.E., Umukoro P.E., Stofell S., Dennerlein J.T., Sorensen G. (2012). Association between Trunk Flexion and Physical Activity in Patient Care Unit Workers. Proceedings of the Human Factors and Ergonomics Society Annual Meeting.

[26] Bootsman R., Markopoulos P., Qi Q., Wang Q., Timmermans A.A., Qi W. (2019). Wearable technology for posture monitoring at the workplace. Int. J. Hum. Comput. Stud..

[27] Jun D., Johnston V., McPhail S.M., O’Leary S. (2019). Are Measures of Postural Behavior Using Motion Sensors in Seated Office Workers Reliable?. Hum. Factors: J. Hum. Factors Ergon. Soc..

[28] Asante B.O., Bath B., Trask C.M. (2018). Trunk posture assessment during work tasks at a Canadian recycling center. Int. J. Ind. Ergon..

[29] Balaguier R., Madeleine P., Rose-Dulcina K., Vuillerme N. (2017). Trunk kinematics and low back pain during pruning among vineyard workers—A field study at the Chateau Larose-Trintaudon. PLoS ONE.

[30] Jakobsen M.D., Sundstrup E., Brandt M., Persson R., Andersen L. (2018). Estimation of physical workload of the low-back based on exposure variation analysis during a full working day among male blue-collar workers. Cross-sectional workplace study. Appl. Ergon..

[31] Marras W.S., Granata K.P., Davis K.G., Allread W.G., Jorgensen M.J. (1997). Spine loading and probability of low back disorder risk as a function of box location on a pallet. Hum. Factors Ergon. Manuf..

[32] Marras W.S., Granata K.P., Davis K.G., Allread W.G., Jorgensen M.J. (1999). Effects of box features on spine loading during warehouse order selecting. Ergonomics.

[33] Schneider S., Lipinski S., Schiltenwolf M. (2006). Occupations associated with a high risk of self-reported back pain: Representative outcomes of a back pain prevalence study in the Federal Republic of Germany. Eur. Spine J..

[34] Beeler N., Roos L., Delves S.K., Veenstra B.J., Friedl K., Buller M.J., Wyss T. (2018). The Wearing Comfort and Acceptability of Ambulatory Physical Activity Monitoring Devices in Soldiers. IISE Trans. Occup. Ergon. Hum. Factors.

[35] Schall M.C., Sesek R., Cavuoto L. (2018). Barriers to the Adoption of Wearable Sensors in the Workplace: A Survey of Occupational Safety and Health Professionals. Hum. Factors J. Hum. Factors Ergon. Soc..

[36] Bergmann J.H., Chandaria V., McGregor A.H. (2012). Wearable and Implantable Sensors: The Patient’s Perspective. Sensors.

[37] Cole G.K., Nigg B.M., Ronsky J.L., Yeadon M.R. (1993). Application of the Joint Coordinate System to Three-Dimensional Joint Attitude and Movement Representation: A Standardization Proposal. J. Biomech. Eng..

[38] Hoogendoorn W.E., Bongers P.M., De Vet H.C.W., Douwes M., Koes B.W., Miedema M.C., Ariëns G.A.M., Bouter L. (2000). Flexion and Rotation of the Trunk and Lifting at Work Are Risk Factors for Low Back Pain. Spine.

[39] Lowe B.D. (2014). Observation-Based Posture Assessment: Review of Current Practice and Recommendations for Improvement.

[40] Mathiassen S.E., Winkel J. (1991). Quantifying variation in physical load using exposure-vs-time data. Ergonomics.

[41] Jansen J.P., Burdorf A., Steyerberg E.W. (2001). A novel approach for evaluating level, frequency and duration of lumbar posture simultaneously during work. Scand. J. Work. Environ. Health.

[42] Coenen P., Kingma I., Boot C.R.L., Twisk J.W.R., Bongers P.M., Van Dieën J.H. (2012). Cumulative Low Back Load at Work as a Risk Factor of Low Back Pain: A Prospective Cohort Study. J. Occup. Rehabil..

[43] Bayoglu R., Galibarov P.E., Verdonschot N., Koopman B., Homminga J. (2019). Twente Spine Model: A thorough investigation of the spinal loads in a complete and coherent musculoskeletal model of the human spine. Med. Eng. Phys..

[44] Kuorinka I., Lortie M., Gautreau M. (1994). Manual handling in warehouses: The illusion of correct working postures. Ergonomics.

[45] Authier M., Lortie M., Gagnon M. (1996). Manual handling techniques: Comparing novices and experts. Int. J. Ind. Ergon..

[46] Burdorf A. (1992). Sources of variance in exposure to postural load on the back in occupational groups. Scand. J. Work. Environ. Health.

[47] Marras W.S., Lavender S.A., Leurgans S.E., Fathallah F.A., Ferguson S.A., Allread W.G., Rajulu S.L. (1995). Biomechanical risk factors for occupationally related low back disorders. Ergonomics.

